# RTS,S vaccination is associated with reduced parasitemia and anemia among children diagnosed with malaria in the outpatient department of a district hospital in rural Malawi

**DOI:** 10.3389/fepid.2022.1039414

**Published:** 2022-11-25

**Authors:** Jacob L. Todd, Hillary M. Topazian, Madalitso Zulu, Pilirani Mafunga, Clement Mapanje, James G. Kaphatika, Maganizo B. Chagomerana, Irving Hoffman, Jonathan J. Juliano, Tisungane Mvalo

**Affiliations:** ^1^Institute for Global Health and Infectious Diseases, University of North Carolina at Chapel Hill, Chapel Hill, NC, United States; ^2^MRC Centre for Global Infectious Disease Analysis, Imperial College London, London, United Kingdom; ^3^University of North Carolina Project Malawi, Lilongwe, Malawi; ^4^Malaria Alert Centre, Kamuzu University of Health Sciences, Blantyre, Malawi; ^5^Department of Medicine, Institute for Global Health and Infectious Diseases, University of North Carolina at Chapel Hill, Chapel Hill, NC, United States; ^6^Division of Infectious Diseases, Department of Medicine, University of North Carolina School of Medicine, Chapel Hill, NC, United States; ^7^Department of Pediatrics, Institute for Global Health and Infectious Diseases, University of North Carolina at Chapel Hill, Chapel Hill, NC, United States

**Keywords:** RTS,S, malaria, vaccine, anemia, parasitemia, parasite density, RTS,S, vaccine, Malawi

## Abstract

The RTS,S/AS01 malaria vaccine was recently approved by the World Health Organization, but real-world effectiveness is still being evaluated. We measured hemoglobin concentration and parasite density in vaccinated and unvaccinated children who had been diagnosed with malaria by rapid diagnostic test (mRDT) in the outpatient department of a rural hospital in Malawi. Considering all mRDT positive participants, the mean hemoglobin concentration among unvaccinated participants was 9.58 g/dL. There was improvement to 9.82 g/dL and 10.36 g/dL in the 1 or 2 dose group (*p* = 0.6) and the 3 or 4 dose group (*p* = 0.0007), respectively. Among a microscopy positive subset of participants, mean hemoglobin concentration of unvaccinated participants was 9.55 g/dL with improvement to 9.82 g/dL in the 1 or 2 dose group (*p* = 0.6) and 10.41 g/dL in the 3 or 4 dose group (*p* = 0.003). Mean parasite density also decreased from 115,154 parasites/μL in unvaccinated children to 87,754 parasites/μL in children who had received at least one dose of RTS,S (*p* = 0.04). In this study population, vaccination was associated with significant improvements in both hemoglobin concentration and parasite density in the setting of real-world administration of the RTS,S/AS01 vaccine.

## Introduction

Significant gains have been made recently in the worldwide fight against malaria (1). The 2021 World Malaria Report states that worldwide malaria cases fell from 81 per 1,000 population at risk in 2000 to 56 in 2019 (1). Despite this progress, the burden of malaria remains a great concern and progress has consistently fallen short of the goals set by the Global Technical Strategy for malaria 2016–2030 (1, 2). New control strategies, such as the RTS,S malaria vaccine, are needed to continue the recent progress. The final results of a phase 3 double-blind, randomized, controlled trial for the RTS,S malaria vaccine were published in 2015 (3). Vaccine efficacy was shown to be 36.3% against clinical malaria and 32.2% against severe malaria (3). Following these results, the vaccine received a positive regulatory assessment from the European Medicines Agency (4), however, the WHO recommended further evaluation to address the concern as to whether the protection could be replicated in the context of administration through routine health systems (5). The Malaria Vaccine Implementation Program (MVIP) was started to pilot RTS,S administration in Malawi, Kenya, and Ghana (5) in 2019. In 2021, based on data from these pilot introductions, the WHO recommended the RTS,S malaria vaccine for children under 5 years of age living in moderate to high malaria transmission areas of sub-Saharan Africa (1, 6).

Given the modest effect of this vaccine, it is imperative to understand the effects of vaccination on malaria associated morbidity and clinical presentation of breakthrough cases. Anemia is common in malaria endemic regions and causes significant mortality and morbidity (7–9). Malaria infection itself is a major cause of anemia and often correlates to increased severity and mortality during malaria infection (9–13). Some studies have also shown that parasite density correlates with disease severity (10, 14, 15), as well as a less efficacious increase in hemoglobin concentration following transfusion for severe malaria anemia (16). Thus, determining what the impact of vaccine on these characteristics of breakthrough infections post vaccination is important.

We conducted a cross-sectional observational study of children aged 6 months to 3 years presenting with malaria rapid diagnostic test (mRDT) positive malaria in order to assess the impact of RTS,S vaccination on anemia and parasitemia at presentation with a breakthrough malaria infection after “real-world” implementation of the vaccine in Malawi through the MVIP. Individuals were enrolled at Mchinji District Hospital, a secondary hospital near both vaccination and non-vaccination clusters for the ongoing World Health Organization RTS,S pilot. We identify measurable benefits on malaria indicators among children who receive the vaccine compared to unvaccinated children.

## Methods

### Study design

We conducted a cross-sectional study of children aged 6 months to 3 years who presented to the outpatient department at Mchinji District Hospital and were diagnosed with malaria by mRDT in order to assess the impact of RTS,S vaccination on malaria associated outcomes such as clinical severity, parasitemia and anemia. Vaccination with RTS,S began in Mchinji District on April 23, 2019. RTS,S vaccination in Malawi is scheduled to be given at 5, 6, 7, and 22 months of age. Vaccination dates were confirmed by inspecting health passports. After informed consent, each caregiver was given a short questionnaire, and the child underwent a targeted physical exam to document signs and symptoms that are consistent with severe malaria. A fingerstick blood sample was collected for analysis of hemoglobin concentration and parasite density. Children were excluded if they had received antimalarial drugs in the previous 28 days. Data were collected from February to May of 2021. Ethical approval was received from the biomedical institutional review board of the University of North Carolina at Chapel Hill, North Carolina USA UNC (20–2153) as well as the Malawi National Health Sciences Research Committee (20/10/2618). Informed consent was obtained from the participants‘ parent or legally accepted guardian prior to any data collection or study specific procedure.

### Laboratory procedures

Blood slides were read independently by at least two qualified microscopists to determine parasite density at the University of North Carolina Project laboratory based at Kamuzu Central in Lilongwe, Malawi. If the first two readings were concordant, the result was taken to be the geometric mean. In the absence of concordance, a third independent reading was done, and the result was the geometric mean between the two geometrically closest readings. All microscopists undergo Glaxo Smith Kline malaria survey external quality assessment exercises every 3 months (17). Point of care hemoglobin was measured by Hemacue Hb 301 at Mchinji District Hospital outpatient department.

### Analysis

Statistical analysis was done using Stata 16.1. Participants were grouped based on the number of vaccine doses they had received. The differences between groups were analyzed using Mann-Whitney U-Test or Fisher's exact test. The significance was set at *p* ≤ 0.05. Linear regression was performed to assess the effects of age on hemoglobin concentration and parasite density. Data from the PEDREF study was used to create a reference hemoglobin for each participant based on age (18). This was done through linear interpolation between the median hemoglobin concentration of a 6-month-old (11.6 g/dL) and the median hemoglobin concentration of a 3-year-old (12.1 g/dL). The PEDREF study created a set of pediatric reference intervals using a database of laboratory results from 12 German tertiary care centers (18).

## Results

### Study population

We enrolled 366 mRDT positive participants. Of the 366, 110 (30%) had evidence of receiving at least one dose of RTS,S/AS01 vaccine in their health passports. A negative malaria microscopy result was found in 136 (37%). Hemoglobin concentration was successfully measured in 364 (99.5%) participants. The date of symptom onset was unavailable for three participants. Summary data by number of vaccine doses are displayed in Table 1. Among the mRDT positive participants, the number of children who presented with signs and symptoms consistent with severe malaria was very low, with only seven participants (2%) citing any severe malaria indicator [6 among unvaccinated and 1 among vaccinated children (*p* = 0.7)]. All children enrolled presented with a Blantyre Coma Score of 5. No children presented with labored breathing, weakness, or pallor. Sensitivity analysis was done to ensure that the low number of participants who presented with signs and symptoms associated with severe malaria did not significantly alter the results of our other analyses. Analyses were repeated after removal of these participants from the data sets. In all cases, the results of this analysis do not differ from the primary analyses.

**Table 1:** Participant characteristics, summarized by number of RTS,S vaccine doses received.

**Variable**	**All** **(*****N*** = **366)**		**0 doses** **(*****N*** = **256)**		**1 dose** **(*****N*** = **4)**		**2 doses** **(*****N*** = **27)**		**3 doses** **(*****N*** = **72)**		**4 doses** **(*****N*** = **7)**	
	***N***	**%**	***N***	**%**	***N***	**%**	***N***	**%**	***N***	**%**	***N***	**%**
**Gender**											
Female	193	52.7	138	53.9	0	0.0	16	59.3	36	50.0	3	42.9
Male	173	47.3	118	46.1	4	100.0	11	40.7	36	50.0	4	57.1
**Microcopy results**											
Microscopy +	230	62.8	162	63.3	3	75.0	18	66.7	41	56.9	6	85.7
Microscopy -	136	37.2	94	36.7	1	25.0	9	33.3	31	43.1	1	14.3
**Age**											
6–12 months	71	19.4	38	14.8	4	100.0	16	59.3	13	18.1	0	0.0
13–18 months	74	20.2	42	16.4	0	0.0	4	14.8	28	38.9	0	0.0
19–24 months	86	23.5	52	20.3	0	0.0	4	14.8	27	37.5	3	42.9
25–30 months	70	19.1	59	23.1	0	0.0	3	11.1	4	5.6	4	57.1
31–36 months	65	17.8	65	25.4	0	0.0	0	0.0	0	0.0	0	0.0
Days to presentation^*^											
<=1	213	58.7	147	57.9	4	100.0	16	59.3	42	59.2	4	57.1
2–3	141	38.8	100	39.4	0	0.0	11	40.7	27	38.0	3	42.9
4–5	9	2.5	7	2.8	0	0.0	0	0.0	2	2.8	0	0.0
Days since vaccination^**^											
<=180 days	48	43.6		NA	4	100.0	16	59.3	21	29.2	7	100.0
>180 days	62	56.4		NA	0	0.0	11	40.7	51	70.8	0	0.0

* Number of days from onset of symptoms to presentation to hospital and enrollment in study.

** Number of days from the most recent dose of RTS,S/AS01 vaccine to presentation to hospital and enrollment in study.

**Table 2:** Study outcomes by vaccination group.

**mRDT** + **participants**					
**Variable**	**All** **(*****N*** = **364)**	**0 doses** **(*****N*** = **255)**	**1 and 2 doses** **(*****N*** = **31)**	**At least 3 doses** **(*****N*** = **78)**	**At least 1 dose** **(*****N*** = **109)**
Mean age (months)	21.04	23.11 (ref)	12.03 (*p* < 0.0001)	17.83 (*p* < 0.0001)	16.18 (*p* < 0.0001)
Mean hemoglobin concentration (g/dL)	9.77	9.58 (ref)	9.83 (*p* = 0.6)	10.36 (*p* = 0.0007)	10.21 (*p* = 0.003)
Age-specific hemoglobin Concentration (g/dL)^*^	11.85	11.89	11.70	11.80	11.77
Mean difference between participant hemoglobin concentration and the age-specific norm (g/dL)	−2.08	−2.30 (ref)	−1.87 (*P* = 0.3)	−1.44 (*p* = 0.0002)	−1.56 (*p* = 0.0004)
**Microscopy** + **participants**					
**Variable**	**All** **(*****N*** = **230)**	**0 doses** **(*****N*** = **162)**	**1 and 2 doses** **(*****N*** = **21)**	**At least 3 doses** **(*****N*** = **47)**	**At least 1 dose** **(*****N*** = **68)**
Mean age (months)	21.89	23.92 (ref)	12.90 (*p* < 0.0001)	18.91 (*p* < 0.0001)	17.06 (*p* < 0.0001)
Mean hemoglobin concentration (g/dL)	9.75	9.55 (ref)	9.82 (*p* = 0.6)	10.41 (*p* = 0.003)	10.23 (*p* = 0.009)
Age-specific hemoglobin Concentration (g/dL)^*^	11.86	11.90	11.72	11.82	11.78
Mean difference between participant hemoglobin concentration and the age-specific norm (g/dL)	−2.11	−2.35 (ref)	−1.89 (*P* = 0.3)	−1.40 (*p* = 0.001)	−1.55 (*p* = 0.002)
Mean parasite density (parasites/μL)	107,194	115,354 (ref)	85,962 (*p* = 0.1)	88,554 (*p* = 0.1)	87,754 (*p* = 0.04)

mRDT, malaria rapid diagnostic test.

* Age-specific norms created by linear interpolation of data from PEDREF study (18).

### Impact of RTS,S vaccination on malaria outcomes among mRDT positive individuals

This analysis included all individuals. As mRDTs do not provide parasitemia estimates, this analysis focused solely on hemoglobin outcomes. A summary of this analysis and the analysis regarding microscopy positive participants that is discussed in the next section can be found in Table 2.

**Figure 1:**
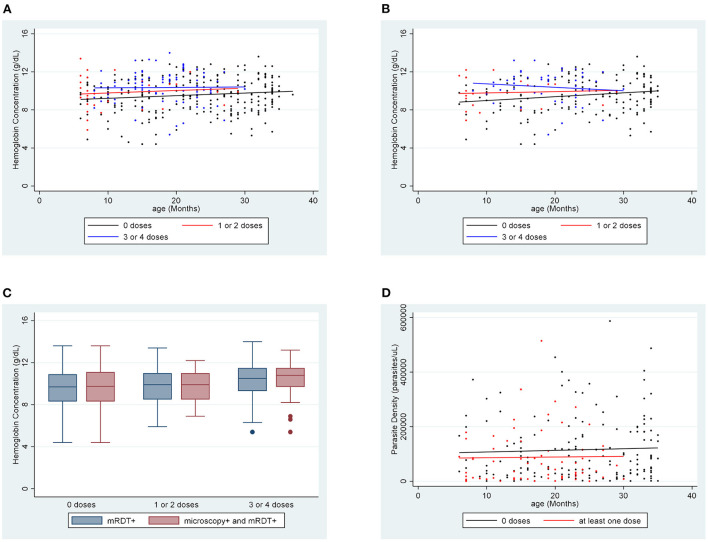
Hemoglobin concentration by age for each vaccination group **(A)** for mRDT + participants and **(B)** for microscopy + participants. **(C)** Comparing the distribution of hemoglobin concentrations among mRDT + and microscopy + participants in each vaccination group. **(D)** Parasite density by age for each vaccination group.

The association between hemoglobin concentration during breakthrough cases of malaria and vaccination status was examined for all 364 participants for which there were successful hemoglobin concentration measurements. The mean hemoglobin concentration of unvaccinated participants was 9.58 g/dL. Hemoglobin concentration improved to 9.83 g/dL in children who had received one or two doses of vaccine (*p* = 0.6) and to 10.36 g/dL in children who received three or four doses of vaccine (*p* = 0.0007). The hemoglobin concentration of each participant was also compared to age-specific norms to account for the known variations of hemoglobin concentration by age within this age group. The mean hemoglobin concentration of unvaccinated participants was 2.30 g/dL below the age-specific norm. The difference was−1.87 and−1.44 g/dL for the one or two dose (*p* = 0.3) and the 3 or 4 dose (*p* = 0.0004) groups, respectively. Regression lines for each vaccination group shows a trend of increasing hemoglobin concentration as the number of vaccine doses increases (Figure 1A). The distribution of hemoglobin concentrations within the data set that includes all mRDT + participants was similar to the distribution of hemoglobin concentrations within the data set that included microscopy confirmed participants (Figure 1C).

Data were analyzed to check for associations between participant sex and hemoglobin concentration. Overall, our sample only slightly favored female participants (53.1%), and no significant differences were found in the percentage of female participants in any of the dose groups. Overall, 28.5% of females were vaccinated with at least one dose, compared to 31.6% of males (*p* = 0.5). Additionally, the mean hemoglobin concentrations among female and male participants within each dose group did not significantly differ (9.73 vs. 9.81 g/dL, *p* = 0.7).

### Impact of RTS,S vaccination on malaria outcomes among microscopy positive individuals

Analysis was also done considering only the 230 participants whose malaria diagnosis was confirmed by microscopy. For this set of data, we included analysis of association between parasite density and vaccination. The mean hemoglobin concentration of unvaccinated participants was 9.55 g/dL. There was improvement to 9.82 g/dL in the one or two dose group (*p* = 0.6) and 10.41 g/dL in the three or four dose group (*p* = 0.003). Hemoglobin concentration in unvaccinated participants was 2.35 g/dL below age-specific norms. Hemoglobin concentration was 1.89 and 1.40 g/dL below norms in the one or two doses (*p* = 0.3) and the three or four doses groups (*p* = 0.001), respectively. For unvaccinated children, the mean parasite density was 115,354 parasites/μL. In children who received at least one dose of vaccine, the mean parasite density fell significantly to 87,754 parasites/μL (*p* = 0.04). Regression lines for both hemoglobin concentration and parasite density for this data set support these trends (Figures 1B,D). These representations also show a trend of improvements in hemoglobin concentration and parasite density with increase in number of vaccine doses.

Again, we assessed the associations between participant sex and our variables of interest. 54.8% of participants were female and 29.4% of female participants had received at least one dose of vaccine, compared to 29.8% of male participants (*p* = 0.9). Mean hemoglobin of female participants was 9.66 g/dL, compared to 9.86 g/dL (*p* = 0.4) among male participants. Mean parasite density among female participants was 104,985 parasites/μL, compared to 109,870 parasites/μL in male participants (*p* = 0.7).

## Discussion

We show an association between vaccination with RTS,S and improvements in hemoglobin levels and parasitemia levels among children with breakthrough cases of malaria who presented to an outpatient department of a district hospital in rural Malawi. These children received the vaccine through routine health systems, outside of a clinical trial. Among mRDT positive individuals in our sample, hemoglobin concentration increased from 9.55 g/dL in the unvaccinated to 10.36 g/dL in those who had received at least 3 doses of vaccine. Among microscopy positive individuals, hemoglobin concentration increased from 9.55 to 10.41 g/dL. Significant improvements in parasite density were also detected with a decrease from 115,354 parasites/μL in the unvaccinated to 87,754 parasites/μL in those who had received at least one dose of vaccine. The association was only seen when comparing unvaccinated children to any children with at least one dose of vaccine. This association was not detected when the one or two dose and the three or four dose groups were separated. The absence of association in these groups may have been due to inadequate sample size.

It was noted that the mean ages of participants in each vaccination dose group varied significantly. This was not unexpected as the vaccine doses are intended to be given at specific ages, 5, 6, 7, and 22 months. A linear regression analysis was conducted to quantify the relationship between age and hemoglobin concentration in this sample. The results show that there was not a statistically significant relationship between age and hemoglobin concentration [*p* = 0.3, risk difference (RD) = 0.011]. Even though the regression analysis did not show a significant effect of age on hemoglobin concentration in our sample, it is well known that hemoglobin concentration tends to increase gradually between the ages of 6 months and 3 years (18). For this reason, a supplemental analysis was conducted to account for this well-known effect by comparing the hemoglobin concentration of each participant to a reference value calculated from external data on age-specific norms. In this supplemental analysis, the mean hemoglobin concentration was below age-specific norms for all groups, however, the differences were less pronounced in the vaccinated groups. This supplemental analysis does not change the conclusion of our primary analysis; however, it is possible that the age discrepancies among our vaccination groups may be leading to an underestimation of the association that we detected.

We acknowledge that the clinical significance of the modest improvements detected in our analysis remains in question. Short-term consequences of anemia can include fatigue, tachycardia, and breathlessness (19), however, as we noted above, the symptoms of the children in this study tended to be mild. Paucity of symptoms suggests that most children in our study were presenting with chronic anemia rather than anemia resulting from a recent rapid reduction in hemoglobin. It has been shown that in Malawi, anemia is significantly more common in children who reported a recent febrile illness (20). Notably, malaria accounts for 30% of visits to outpatient medical centers in Malawi (21). Chronic anemia in children is associated with a number of serious long-term sequelae including growth impairment, developmental delays, and decreased school performance (19), but it is difficult to determine whether these consequences are related to the anemia itself or the underlying cause. A meta-analysis regarding anemia in African children did show a 24% reduction in risk of death for each 1 g/dL increase in hemoglobin concentration, but most of the children in this analysis had much more severe anemia than the children in our study (22). Many studies have shown a correlation between lower hemoglobin concentration and increased severity during episodes of malaria (10–13), but we are not able to draw strong comparisons between these studies and our data since we did not enroll children with severe disease.

The usefulness of measuring parasite density in determining malaria severity is somewhat controversial. Some studies have shown an association between parasite density and disease severity (10, 15), while other studies have not (14, 23). One large study that enrolled hospitalized children across 9 African countries did show an association between high parasite density and risk of death, but only at very high levels, above 440,000 parasites/μL (10). Only 4 children in our study had parasite density greater than this mark.

It should be stressed that several potentially important confounders such as socioeconomic status, caregiver education, and whether participants slept under mosquito nets were not included in this study. Therefore, we are only able to assess marginal bivariate associations, and cannot determine any causal links. In our sample, lower parasite density and higher hemoglobin concentration were associated with vaccination during breakthrough cases of disease. It may be beneficial to further explore these associations in the future. We were not able to draw any conclusions regarding an association between vaccination and likelihood of presenting with less severe disease due to low numbers of participants with severe disease indicators. Children who presented to the hospital with severe disease were typically admitted directly to the inpatient ward after triage, bypassing our data collection point in the outpatient department. Our study was limited to the outpatient setting and not inpatients with more severe disease. A future study with a larger sample size and a more robust data collection team may be able to detect such an association.

This is among the first published results to show a positive effect of RTS,S vaccination administered outside of a clinical trial. Even though we cannot demonstrate a causal link, it is feasible that vaccine induced immunity led to lower parasite density during breakthrough infection. We can also hypothesize that improvements in hemoglobin concentration could be secondary to fewer episodes of malaria in vaccinated children. It could be beneficial to explore these associations in more detail. We recommend that future studies enroll children including those with severe disease to measure the full magnitude of the associations. Of particular interest would be to explore whether vaccination results in a reduced likelihood of blood transfusion in cases of severe malaria.

## Data availability statement

The raw data supporting the conclusions of this article will be made available by the authors, without undue reservation.

## Ethics statement

The studies involving human participants were reviewed and approved by Biomedical Institutional Review Board of the University of North Carolina at Chapel Hill, Malawi National Health Sciences Research Committee. Written informed consent to participate in this study was provided by the participants' legal guardian/next of kin.

## Author contributions

JT contributed to conceptualization, design, data collection, analysis, and writing. HT and MC contributed to design, analysis, and writing. MZ, PM, and JK contributed to data collection and writing. CM contributed to writing. IH, JJ, and TM contributed to conceptualization, design, analysis, and writing. All authors contributed to the article and approved the submitted version.

## Funding

This work was funded by UJMT Fogarty Global Health Fellowship Program and the Fogarty International Center within the National Institutes of Health (D43-TW009340).

## Conflict of interest

The authors declare that the research was conducted in the absence of any commercial or financial relationships that could be construed as a potential conflict of interest.

## Publisher's note

All claims expressed in this article are solely those of the authors and do not necessarily represent those of their affiliated organizations, or those of the publisher, the editors and the reviewers. Any product that may be evaluated in this article, or claim that may be made by its manufacturer, is not guaranteed or endorsed by the publisher.
